# Enhancing Surgical Planning with AI-Driven Segmentation and Classification of Oncological MRI Scans

**DOI:** 10.3390/s26010323

**Published:** 2026-01-04

**Authors:** Alejandro Martinez Guillermo, Juan Francisco Zapata Pérez, Juan Martinez-Alajarin, Alicia Arévalo García

**Affiliations:** 1 Escuela Tecnica Superior de Ingenieria Industrial, Campus Muralla del Mar, Universidad Politecnica de Cartagena Member of European University of Technology EUT+, C/Doctor Fleming, s/n, 30202 Cartagena, Spain; juan.zapata@upct.es (J.F.Z.P.); juanc.martinez@upct.es (J.M.-A.); 2 Cella Medical Solutions, 30100 Murcia, Spain; aliciaarevalo@cellams.com

**Keywords:** artificial intelligence, 3D reconstruction, image-derived information, Dice score improvement, surgical planning, anatomical segmentation, oncological imaging, magnetic resonance imaging (MRI)

## Abstract

This work presents the development of an Artificial Intelligence (AI)-based pipeline for patient-specific three-dimensional (3D) reconstruction from oncological magnetic resonance imaging (MRI), leveraging image-derived information to enhance the analysis process. These developments were carried out within the framework of Cella Medical Solutions, forming part of a broader initiative to improve and optimize the company’s medical-image processing pipeline. The system integrates automatic MRI sequence classification using a ResNet-based architecture and segmentation of anatomical structures with a modular nnU-Net v2 framework. The classification stage achieved over 90% accuracy and showed improved segmentation performance over prior state-of-the-art pipelines, particularly for contrast-sensitive anatomies such as the hepatic vasculature and pancreas, where dedicated vascular networks showed Dice score differences of approximately 20–22%, and for musculoskeletal structures, where the model outperformed specialized networks in several elements. In terms of computational efficiency, the complete processing of a full MRI case, including sequence classification and segmentation, required approximately four minutes on the target hardware. The integration of sequence-aware information allows for a more comprehensive understanding of MRI signals, leading to more accurate delineations than approaches without such differentiation. From a clinical perspective, the proposed method has the potential to be integrated into surgical planning workflows. The segmentation outputs were converted into a patient-specific 3D model, which was subsequently integrated into Cella’s surgical planner as a proof of concept. This process illustrates the transition from voxel-wise anatomical labels to a fully navigable 3D reconstruction, representing a step toward more robust and personalized AI-driven medical-image analysis workflows that leverage sequence-aware information for enhanced clinical utility.

## 1. Introduction

Cancer remains one of the most pressing global health challenges, characterized by the uncontrolled growth and spread of abnormal cells that can invade surrounding tissues and metastasize to distant organs. According to the most recent GLOBOCAN estimates, more than 20 million new cancer cases and 9.7 million cancer-related deaths were reported worldwide in 2022, with projections indicating an increase to over 35 million new cases annually by 2050 [1]. This rising burden is influenced by population growth, aging, lifestyle factors, and environmental exposures.

At the level of developed countries, similar trends are observed across multiple tumor types. Although incidence and mortality vary by region and socioeconomic context, factors such as obesity, smoking, alcohol consumption, sedentary lifestyles, and reproductive patterns play a significant role in cancer epidemiology. The increasing incidence of early-onset cancers further underscores the need for more effective diagnostic and treatment strategies.

Early detection remains a cornerstone for improving cancer outcomes, as timely diagnosis is associated with higher survival rates, reduced treatment-related morbidity, and improved quality of life. International health organizations emphasize that delays in diagnosis significantly compromise treatment success, whereas early identification enables more effective and less invasive therapeutic interventions.

Within oncological management, surgical treatment continues to play a central role, particularly for solid tumors. Successful surgical outcomes depend heavily on accurate preoperative planning, which requires precise knowledge of tumor extent and its spatial relationship with surrounding anatomical structures. In this context, medical imaging is indispensable. Among imaging modalities, magnetic resonance imaging (MRI) offers excellent soft-tissue contrast, multiplanar capabilities, and the absence of ionizing radiation, making it especially valuable for oncological applications in the brain, abdomen, pelvis, and musculoskeletal system.

Despite these advantages, the manual interpretation, classification, and segmentation of MRI scans remain time-consuming and subject to inter- and intra-observer variability. Radiological workflows rely extensively on expert knowledge to identify relevant anatomical structures and differentiate tissue characteristics across MRI sequences, a process that becomes increasingly challenging with the growing volume and complexity of imaging data.

Artificial intelligence (AI), particularly deep learning (DL), has emerged as a powerful tool to address these challenges. Convolutional neural networks have demonstrated strong performance in medical image classification and segmentation tasks, enabling automated extraction of hierarchical features directly from imaging data. Architectures such as U-Net and its derivatives, including nnU-Net, have established themselves as reference methods for biomedical image segmentation due to their adaptability and robustness across diverse datasets.

However, many existing AI-based MRI pipelines focus primarily on segmentation while overlooking the explicit role of MRI sequence type in image appearance and contrast. In clinical practice, differences between T1- and T2-weighted sequences significantly affect tissue visualization and anatomical delineation, suggesting that sequence-aware approaches may provide more accurate and clinically meaningful results.

In this work, we propose an integrated AI-driven pipeline for oncological MRI analysis that explicitly incorporates MRI sequence classification as a preliminary step. The framework automatically classifies MRI volumes into T1- or T2-weighted sequences and subsequently applies sequence-specific and general deep learning segmentation models to generate three-dimensional anatomical reconstructions suitable for surgical planning.

The main contributions of this study include: (i) the development of an automatic MRI sequence classification module based on deep learning, (ii) the design and evaluation of sequence-specific and general segmentation networks for clinically relevant abdominal structures, and (iii) the integration of the resulting segmentations into a three-dimensional visualization environment to support surgical planning workflows.

In the remainder of this work, we first review prior research on artificial intelligence applied to oncological MRI, with emphasis on classification and segmentation strategies, which is provided in Section 2. Section 3 describes the methodology adopted in this study, including dataset preparation, model architectures, training procedures, and implementation details. The experimental results are presented next in Section 4, covering both quantitative evaluations and qualitative analyses of the model’s assessments by clinical experts. Next, in Section 5, we discuss the main findings and their implications. Section 6 summarizes the limitations of this study and outlines future work directions. Finally, in Section 7, we conclude the manuscript by highlighting the main contributions.

## 2. Related Work

This section reviews methods for enhancing surgical planning with AI-driven segmentation and classification of oncological MRI scans. We organize prior work into six thematic areas:

(i) Classical and radiomics-based approaches;
(ii) Deep learning approaches for segmentation;
(iii) Deep learning approaches for classification;
(iv) Hybrid pipelines combining classification and segmentation;
(v) Learning with limited labels and domain shift;
(vi) Clinical integration, uncertainty, and evaluation.

The first category, classical and radiomics-based approaches, formed the foundation of early clinical pipelines for oncological MRI analysis. Atlas-based registration methods demonstrated strengths in anatomically constrained environments but showed sensitivity to registration errors and anatomical variability [2]. Graph cut formulations enabled efficient global energy minimization for segmentation but required careful hand-crafting of energy terms and parameter tuning [3]. These methods, while foundational, faced limitations in reproducibility and scalability within large-scale surgical workflows.

The emergence of radiomics addressed some limitations by extracting hand-crafted intensity, texture, and shape features for classical machine learning classifiers. Aerts et al. demonstrated the prognostic value of engineered features across multiple cancers [4]. However, feature stability across scanners and sequences remained a significant challenge, limiting deployment in heterogeneous MRI cohorts. These approaches fundamentally lacked the adaptability and representation learning capabilities of modern deep learning methods.

The second category, deep learning approaches for segmentation, was revolutionized by the introduction of the U-Net architecture, which combined multi-scale contracting and expanding paths with skip connections [5]. Its extensions to 3D volumes proved essential for oncological MRI applications. Çiçek et al. introduced 3D U-Net for volumetric segmentation [6], while Milletari et al. proposed V-Net with Dice-based loss to address class imbalance in lesion segmentation [7]. The nnU-Net framework further advanced the field by automating preprocessing, network configuration, and postprocessing [8], demonstrating that principled heuristics could rival bespoke designs. Architectural refinements including U-Net++ with dense skip connections [9] and Attention U-Net with spatial gating mechanisms [10] improved boundary precision and sensitivity to small, low-contrast lesions typical in oncologic MRI.

The third category, deep learning approaches for classification, brought significant advances to MRI sequence identification and tumor characterization. ResNet architectures stabilized training in very deep networks through residual connections [11], while DenseNet improved feature reuse and parameter efficiency [12]. EfficientNet introduced compound scaling to balance depth, width, and resolution [13], particularly beneficial for high-resolution MRI data. More recently, Vision Transformers [14] and Swin Transformers [15] modeled long-range dependencies through self-attention mechanisms, showing promising results for medical image classification tasks.

The fourth category, hybrid pipelines and multi-stage approaches, offers a practical strategy for handling heterogeneous MRI cohorts by first classifying acquisition sequences or tumor categories before performing segmentation. Litjens et al. documented consistent performance gains from such modular designs in their comprehensive survey of deep learning in medical imaging [16]. Within neuro-oncology, the BraTS challenge catalyzed development of cascaded and multi-branch networks that integrate sequence-level cues with subregion segmentation [17,18]. These approaches demonstrated the benefits of leveraging complementary sequences (e.g., FLAIR for edema, T1c for enhancing core) but often lacked explicit sequence classification modules to normalize inputs prior to segmentation.

These approaches demonstrated the benefits of leveraging complementary sequences but often operated under the assumption of standardized input protocols. The critical step of explicit sequence classification to normalize inputs prior to segmentation—particularly vital in multi-center surgical settings with inherent protocol variability—remains underexplored, a gap our pipeline specifically addresses.

The fifth category, learning with limited labels and domain adaptation, addresses the fundamental challenge of label scarcity in oncology MRI. Semi-supervised and self-supervised approaches have shown promise in mitigating this limitation. Bai et al. utilized weak supervision and shape priors for cardiac MRI segmentation [19], while Chaitanya et al. employed contrastive learning to develop robust representations from limited annotations [20]. Domain adaptation methods have specifically addressed the challenge of distribution shift across institutions. Kamnitsas et al. introduced unsupervised domain adaptation for lesion segmentation [21], and Karani et al. proposed task-driven image harmonization to reduce inter-site variability [22]. These techniques remain under-utilized in surgical planning-grade studies, where their potential impact is magnified by the prohibitive cost of expert annotation and the critical need for model robustness across institutional boundaries in multi-center evaluations aligned with clinical endpoints.

The last category, clinical integration and uncertainty quantification, emphasizes that calibrated uncertainty estimates and robust evaluation metrics are essential for clinical deployment. Kendall et al. decomposed predictive uncertainty into aleatoric and epistemic components, demonstrating improved reliability for segmentation tasks [23]. Taha and Hanbury analyzed metric behavior and pitfalls in 3D segmentation, recommending complementary measures for surgical planning validation [24]. Despite these advances, uncertainty quantification and calibration are not routinely reported in segmentation studies. Furthermore, while technical performance metrics dominate the literature, few works deliver modular, auditable pipelines that output patient-specific 3D assets directly consumable by surgical planning platforms—a crucial translation gap identified in comprehensive reviews of medical AI deployment [25].

In summary, existing AI-based pipelines for oncological MRI analysis have demonstrated remarkable performance in controlled research settings, yet several limitations persist. Many approaches fuse sequences without explicit classification and normalization, show limited multi-center generalization, underutilize semi- or self-supervised methods, provide insufficient uncertainty quantification, and rarely offer modular, auditable pipelines suited for clinical integration. To address these challenges, our work introduces a modular pipeline that classifies MRI sequences prior to segmentation, employs robust NN-based segmentation with standardized preprocessing and postprocessing, and produces calibrated, auditable results ready for clinical deployment. By bridging these gaps, our approach advances the translation of technical innovation into clinical applicability for oncological MRI surgical planning.

## 3. Methodology

**Figure 1:**
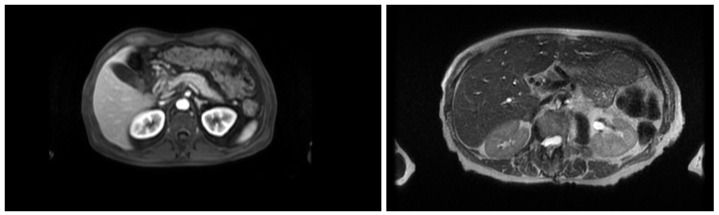
The (**left**) picture shows a T1-weighted MRI scan, where fat-rich tissues appear hyperintense, providing clear anatomical boundary definition. The (**right**) picture shows T2-weighted MRI scan, where fluid-rich structures such as cerebrospinal fluid appear hyperintense, enhancing contrast in edema, inflammation, and other fluid-related pathologies.

This section describes the dataset, model architecture, and training procedures used for MRI sequence classification (T1 vs. T2) and anatomical segmentation as part of an AI pipeline for surgical planning. In the stage of classification, the study was conducted in three stages of training, progressively refining the dataset and model configuration. The dataset was refined across three training stages: an initial set of 363 volumes, a filtered and expanded set of 2286 volumes, and a third training stage focusing on extended epochs to optimize performance. A ResNet18 architecture was adapted for volumetric inputs and binary classification. To support this task, the inherent contrast differences between T1- and T2-weighted MRI were leveraged: in T1-weighted images, fat-rich tissues appear hyperintense, providing clear anatomical boundary definition, whereas in T2-weighted images the fluid-rich structures such as cerebrospinal fluid appear hyperintense, enhancing contrast in edema, inflammation, and other fluid-related pathologies, as shown in Figure 1. In contrast, fluid-containing structures present higher signal intensity, making them more suitable for highlighting pathological or inflammatory conditions. Next, a segmentation stage was implemented, which employed a modular nnU-Net framework with networks specialized in anatomical subsets including major organs, vasculature, musculoskeletal structures, and tumors. Training used the Adam optimizer with carefully selected batch sizes, learning rates, and epochs adjusted per dataset and task. Model performance was evaluated using Dice coefficient, Intersection over Union, and Hausdorff distance. The subsequent subsections present a comprehensive and detailed explanation.

### 3.1. Dataset

The dataset used in this study consists of clinical MRI images stored in Cella’s internal PACS system, covering brain, thoracic, abdominal, and pelvic regions. The study was conducted in three training stages, progressively refining both the dataset and the model configuration.

As an initial proof-of-concept, a preliminary dataset of 363 MRI images (174 T1-weighted and 189 T2-weighted) was assembled to establish a first contact with the classification task. This dataset was split in a stratified manner: 70% for training, 10% for validation, and 20% for testing, preserving class proportions. Each 3D image acquisition was pre-processed to extract representative axial slices from the central region of each volume, capturing the most relevant anatomical information while avoiding peripheral slices with potential artifacts. Intensities were rescaled to [0–1], and data augmentation—including rotation, translation, noise addition, brightness adjustment, and blurring—was applied.

To increase diversity and representativeness, in our second approach, the dataset was expanded and filtered using strict inclusion criteria. Only volumes acquired in the axial plane with at least 30 slices were retained. Multivolume scans containing multiple acquisitions within a single file were excluded. Initial manual inspection using 3D Slicer verified slice count, orientation, and single-series compliance. Subsequently, a custom script automated the analysis of metadata to confirm axial orientation and detect multivolume cases. After filtering, the final dataset comprised 2286 MRI volumes, with 1588 T1-weighted and 698 T2-weighted images. The dataset was stratified into 1600 training images, 229 validation images, and 457 test images, maintaining balanced representation of both classes. Each volume continued to use 10 central slices for model input. Table 1 shows the distribution of the dataset in each approach.

There were no major modifications to the dataset in the third approach. The dataset for the third training remained the same (2286 volumes, same splits), as the focus was on further optimizing model performance by fine-tuning the network rather than expanding or improving the dataset.

Given the objective of developing a modular and progressive segmentation pipeline, a specialized dataset was prepared, consisting of MRI images—T1-weighted images, T2-weighted images, and a combined MR dataset including both sequences—and corresponding anatomical blocks of clinical relevance serving as ground truth. This approach enables the training of specialized networks on specific subsets of anatomical regions, facilitating the creation of a progressive and optimized segmentation workflow. The selection of structures was guided by both clinical importance and the necessity of accurate delineation in procedures such as tumor resection or liver transplantation planning, where precise identification of key anatomical structures is critical. Table 2 summarizes the selected anatomical structures, grouped by clinical and anatomical relevance. The images and annotations were provided and carefully reviewed by medical imaging technicians and radiologists to ensure accuracy. This organization facilitated training of specialized networks and allowed comparison between networks trained on single sequences (T1w, T2w) versus the combined dataset. A specialist network was created for each MRI sequence and anatomical group, resulting in a total of 9 segmentation networks, given the three anatomical groups and three types of MRI sequences.

The development of the segmentation networks followed a unified methodology aimed at identifying and prioritizing anatomically and clinically relevant structures. A general workflow was established for dataset preparation, label definition, and model configuration, which was subsequently applied across all trained networks.

During data preparation, custom scripts were implemented to traverse the complete segmentation masks and retain only the regions corresponding to the target anatomical structures (e.g., vascular systems, abdominal organs, or other specific components depending on the network), discarding all irrelevant tissues. This preprocessing step produced focused datasets in which each image was exclusively paired with masks of the structures of interest, minimizing interference from surrounding anatomy.

It was also observed that requiring all target labels to be present simultaneously drastically reduced the number of usable samples. To overcome this limitation, an additional script was implemented to relax this constraint, allowing the inclusion of cases containing at least one of the target structures. This adjustment significantly increased dataset size and variability, improving model robustness and generalization.

The segmentation networks were implemented using the nnU-Net framework, leveraging its ability to adapt automatically to dataset properties such as patch size, voxel spacing, and number of classes. The architecture followed a 3D U-Net structure with encoder–decoder pathways, residual connections, and softmax-based multi-class output layers. Each network was trained to segment up to three classes (portal, venous, arterial vasculatures), with the output channel dimension adapted accordingly. Skip connections between the encoder and decoder ensured the preservation of fine-grained spatial details, which is critical for vascular structures due to their thin and branching morphology.

### 3.2. Ethical Approval, Data Anonymization, and Acquisition Characteristics

All data were fully anonymized prior to analysis in accordance with the General Data Protection Regulation (GDPR), and no personally identifiable information was accessible to the researchers. All imaging data used in this study were retrospectively collected from Cella Medical Solutions’ internal PACS system, which aggregates clinical MRI studies from more than 600 healthcare centers across different countries of America and Europe. This multicenter and multinational composition increases dataset heterogeneity and better reflects real-world clinical variability. The study population mainly consisted of adult patients, with an estimated sex distribution of approximately 70% male and 30% female subjects, which is consistent with the demographic profile commonly observed in abdominal oncological imaging cohorts.

MRI examinations were acquired as part of routine clinical practice using scanners from multiple vendors; approximately 45% of the MRI studies were acquired using Siemens systems, 30% using GE scanners, and 15% using Philips scanners, while the remaining 10% corresponded to other vendors. Acquisition parameters and imaging protocols varied depending on clinical indication, anatomical region, scanner configuration, and institutional imaging practices. This variability reflects standard clinical conditions rather than controlled research acquisitions. The majority of MRI studies corresponded to oncological examinations of the abdominopelvic region. The most prevalent clinical indications included abdominal and abdominopelvic cancers, predominantly hepatobiliary, pancreatic, colorectal, and retroperitoneal malignancies. A smaller proportion of cases included non-oncological indications such as inflammatory processes, benign lesions, or postoperative and treatment follow-up studies. The dataset included adult patients with an age range between approximately 18 and 70 years. Regarding geographic origin, the cohort was composed of imaging studies acquired in healthcare centers from both Europe and the American continent, with an estimated distribution of approximately 60% European centers and 40% centers from North and South America. Only aggregated demographic information was available for analysis, and no direct patient identifiers were accessible at any stage of the study.

Given the retrospective nature of the study and the use of anonymized and aggregated imaging data, the requirement for formal institutional review board approval and individual patient consent was waived in accordance with applicable institutional policies and national regulations.

### 3.3. Model Architecture

For sequence classification, a ResNet18 convolutional neural network was employed due to its balance between depth and computational efficiency. ResNet18 was chosen over deeper variants due to its balance between representational capacity and computational efficiency, particularly important given our dataset size and clinical deployment constraints. Its flexible design facilitated integration with data loading, preprocessing, and augmentation pipelines. The network was adapted to handle volumetric data: the first convolutional layer was modified to accept 10-channel inputs, corresponding to the 10 central slices of each volume, and the final fully connected layer was adjusted to have two output neurons for binary classification (T1 vs. T2).

Prior to designing the segmentation networks, an exploratory analysis of the dataset was performed to assess the availability of anatomical structures across the three imaging categories (T1, T2, and MR). A modular segmentation strategy was adopted, with networks specialized in subsets of anatomical structures. A dedicated analysis script was implemented to traverse the dataset, identify the presence of annotated structures in each case, and generate distribution plots for each imaging category. Structures of higher clinical relevance were prioritized during the initial stages of network training. In particular, vascular structures were segmented first using a specialized vascular network, given their critical role in surgical planning and their impact on subsequent organ delineation. Once the vasculature was accurately defined, the segmentation of abdominal organs was performed, followed by musculoskeletal components such as bones and muscles. This hierarchical strategy ensured that anatomically and functionally interdependent regions were processed in a clinically meaningful order, improving overall segmentation consistency and precision.

Modularity was a deliberate design principle, allowing each network to specialize in a subset of structures and enabling the output of one network to guide or constrain the next. This sequential refinement enhanced segmentation accuracy in neighboring regions while reducing computational load. Each network operated on 3D patches extracted from T1, T2, or combined MR datasets and generated corresponding 3D segmentation masks. The modular workflow facilitated convergence, optimized resource usage, and supported a progressive, anatomically coherent reconstruction of the human body.

### 3.4. Training Procedure

In our initial approach, a ResNet18 model was trained on our initial dataset of 363 MRI images (174 T1-weighted and 189 T2-weighted). This dataset was split in a stratified manner to preserve class proportions of the 363-image dataset with a batch size of 1, for 70 epochs, and a learning rate of 0.001 using a Stochastic Gradient Descent (SDG) optimizer. Preprocessing and data augmentation were applied to each image as described above, to enhance model robustness and generalization.

Once our dataset was expanded, we conducted a second training using the filtered dataset of 2286 volumes, and then the model was retrained with updated hyperparameters: batch size 16,100 epochs, and learning rate 0.001 and using, again, an SDG optimizer. Stratified splitting ensured balanced representation of T1 and T2 images. This allowed the model to generalize better and capture more robust patterns across a larger dataset.

In the next approach and in order to further stabilize performance, a third training was conducted on the same 2286-volume dataset, keeping the same ResNet18 architecture, batch size, and learning rate, but increasing the number of epochs to 200. The input continued to consist of 10 central slices, and the train/validation/test splits remained unchanged. This stage aimed to refine the model, ensuring convergence and optimal accuracy for T1/T2 classification.

For segmentation, networks nnU-Net were trained using stratified splits of the dataset for training, validation, and testing, ensuring balanced representation of all structures in each subset. The SDG optimizer was employed with a learning rate of 0.001. Batch sizes were adjusted according to the patch size and GPU memory availability.

The training strategy was designed to exploit the modular structure of the segmentation pipeline. Each specialized network was trained independently on a reduced set of anatomical structures, using the nnU-Net’s standardized configuration of patch-based 3D convolutions, encoder–decoder architecture, and softmax output layers. The sequential ensemble approach of networks enabled refinement of the segmentation process: predictions from earlier networks were incorporated as priors for later stages, allowing subsequent models to adjust their focus depending on the confidence of previous outputs. This design not only reduced computational requirements but also improved anatomical consistency across segmentations.

To investigate the impact of modality-specific learning versus a pooled approach, nine separate networks were trained: (i) a T1-specific network for anatomical structures, (ii) a T2-specific network for anatomical structures, and (iii) a general MR network combining T1 and T2 images without prior stratification for anatomical structures. For each of these three main networks, an additional set of three specialized models was trained to segment different anatomical groups, resulting in a total of nine trained models. This experimental setup enabled the evaluation of whether modality specialization improved segmentation performance compared to a pooled training strategy.

Each network was initially trained for 1000 epochs with a constant learning rate of 0.01. Table 3 summarizes the segmentation network architecture. A second series of experiments extended the training to 1500 epochs, with an increased initial learning rate of 0.03, proportional to the number of labels present in the dataset. Despite the extended training time and adjusted learning rate, performance differences were minimal. For example, in the MR network, the Dice similarity coefficient (DSC) on the test set was 0.7774 after 1000 epochs and 0.7719 after 1500 epochs, indicating no significant improvement with prolonged training.

During training, data augmentation was applied, including random rotation, scaling, elastic deformation, Gaussian noise, and intensity adjustments, to increase model robustness and compensate for limited data. For sequential networks, masks predicted by previous networks were incorporated as additional inputs, allowing subsequent networks to focus on refining predictions in relevant regions. Model performance was evaluated using standard metrics such as Dice coefficient, Intersection over Union (IoU), and Hausdorff distance. Hyperparameters and augmentation strategies were iteratively adjusted based on validation performance to ensure stable convergence and reliable generalization.

### 3.5. Implementation Details

The development of the MRI sequence classification and segmentation pipeline leveraged a variety of specialized libraries, frameworks, and tools to ensure efficient processing, model training, and evaluation. The core implementation was carried out in Python (v3.9), taking advantage of its extensive ecosystem for scientific computing and deep learning.

For deep learning model development, the PyTorch (v1.13) framework was employed due to its flexibility in designing custom neural network architectures, dynamic computation graphs, and robust support for GPU acceleration.

Medical image processing and manipulation were facilitated by tools like SimpleITK (v2.2.1) and Nibabel (v5.1.0), enabling loading, resampling, intensity normalization, and conversion between different medical imaging formats. Data augmentation was implemented using TorchIO (v0.19.1). and custom scripts to apply rotations, translations, elastic deformations, Gaussian noise, and intensity adjustments, ensuring robust model training.

For visualization and quality control, Matplotlib (v3.7.1), Seaborn (v0.12.2), and 3D Slicer (v4.11) were employed to inspect MRI volumes, overlay segmentation masks, and verify preprocessing steps. Finally, data handling, dataset organization, and experiment tracking were managed using Pandas (v2.0.3) and NumPy (v1.24.4), allowing structured storage of image paths, labels, and metadata for reproducible experimentation.

## 4. Results

### 4.1. T1 and T2 MRI Classification

**Figure 2:**
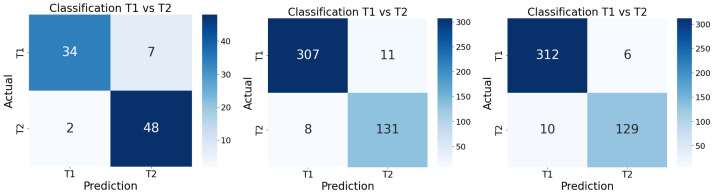
Confusion matrix for the first training stage (T1 vs. T2) on test set (**left**), second training stage (T1 vs. T2) on test set (**middle**), and third training stage (T1 vs. T2) on test set (**right**). Confusion matrix (**left**) shows good classification performance, with only 9 (9.89%) misclassified images. Confusion matrix (**middle**) shows improved classification performance, with only 19 (4.16%) misclassified images. Finally, confusion matrix (**right**) shows further improved classification performance, with only 16 (3.5%) misclassified images. The model is therefore considered the final version for the task of automatic classification of T1- and T2-weighted MRI images on the dataset used.

The first training stage involved the ResNet18 model trained on the initial dataset of 363 MRI volumes. After completing training, the model was evaluated on the test set, yielding the confusion matrix on the left of Figure 2 and the performance metrics summarized in Table 4. The model achieved a global accuracy of 0.90 with a support of 41 T1-weighted images and 50 T2-weighted images on the test set. T1-weighted images had a precision of 0.94, with a recall of 0.83 and an F1-score of 0.88, while T2-weighted images had a precision of 0.87, with a recall of 0.96 and an F1-score of 0.91 The confusion matrix indicates that the model correctly classified 34 out of 41 T1-weighted images and 48 out of 50 T2-weighted images, with a total of 9 misclassifications, including 7 T1-weighted images misclassified as T2-weighted and 2 T2-weighted images misclassified as T1-weighted.

Analysis of these metrics reveals that, while the model achieved high performance for T2-weighted images, its performance on T1-weighted images was notably lower, with a recall of 0.83 and an F1-score of 0.88. This imbalance indicates that the model did not robustly capture the distinguishing features between the two classes. This limitation may be partly attributed to the relatively small size of the training dataset and the potential heterogeneity of the images. Additionally, the model’s performance on T2-weighted images was generally higher, with a precision of 0.87, a recall of 0.96, and an F1-score of 0.91.

Based on these preliminary results, a promising strategy to enhance model performance is to substantially expand the dataset by incorporating a larger number of images from both classes. Increasing the size and diversity of the dataset enables the model to learn more generalizable and robust features, thereby improving its ability to generalize and achieving better classification accuracy on unseen data. These insights informed the design of the subsequent training stages, which emphasized dataset expansion (as explained above in Section 3.1) along with the refinement of preprocessing and augmentation techniques.

The second training stage employed the expanded and filtered dataset of 2286 MRI volumes. After completing training, the model was evaluated on the test set, which yielded the results presented in the confusion matrix in the middle of Figure 2 and Table 4. The model achieved a global accuracy of 0.96 with a support of 318 T1-weighted images and 139 T2-weighted images on the test set. T1-weighted images had a precision, recall and F1-score of 0.97, while T2-weighted images had a precision of 0.92, with a recall of 0.94 and an F1-score of 0.93. The confusion matrix indicates that the model correctly classified 309 out of 318 T1-weighted images and 131 out of 139 T2-weighted images, with a total of 19 misclassifications, including 11 T1-weighted images misclassified as T2-weighted and 8 T2-weighted images misclassified as T1-weighted. Therefore, the model demonstrated significant improvement compared to the first training stage, likely due to the increased dataset size and diversity. This is due to the model effectively learning to distinguish between the two sequences, mainly because of the increased number of T1-weighted images in the dataset.

**Figure 3:**
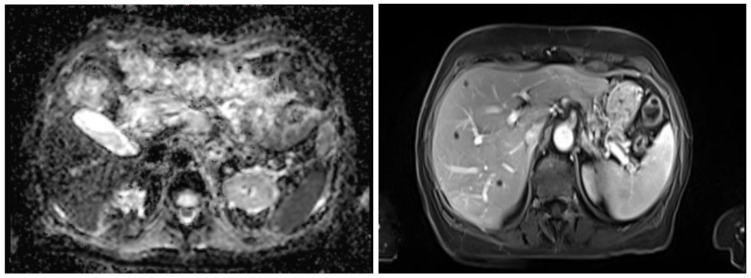
Example of a misclassified T1/T2 MRI image affected by noise or low contrast. (**Left**): Noisy image. (**Right**): Clear image (it is not the same image as the left one).

A manual review of misclassified images revealed that many errors were associated with atypical characteristics, including low-contrast regions, image artifacts, or noise, as shown in Figure 3. These characteristics were primarily observed in a limited subset of clinical cases, typically corresponding to challenging acquisition conditions rather than standard MRI studies. In particular, artifacts and noise were more frequently encountered in scans affected by patient motion, reduced acquisition time, suboptimal signal-to-noise ratio, or imaging performed in complex clinical contexts. Overall, these atypical acquisition conditions were estimated to affect approximately 5% of the analyzed MRI studies. This post-training analysis suggests potential improvements in preprocessing strategies or the incorporation of additional data augmentation techniques to increase model robustness against complex and non-ideal acquisition scenarios. While such atypical characteristics are not dominant in routine clinical MRI, they represent realistic edge cases that can negatively impact automated classification performance. In particular, images with significant noise or low contrast were more prone to misclassification, indicating that enhancing image quality or incorporating noise-robust features during training could further improve classification accuracy.

Overall, the second training stage demonstrates a substantial improvement over the first, with higher precision, recall, and F1-scores for both classes, indicating that dataset expansion and careful filtering significantly enhanced the model’s generalization capabilities. Therefore, a further expansion of the dataset and an increased number of training epochs allowed the model to learn complex patterns from the expanded dataset, resulting in improved performance on the test set.

The third training stage utilized the same expanded dataset of 2286 MRI volumes, with the primary modification being an increase in the number of training epochs from 100 to 200. This adjustment aimed to further refine the model’s learning and improve its classification performance. After completing training, the model was evaluated on the test set where its confusion matrix is shown on the right of Figure 2, and the detailed performance metrics are summarized in Table 4. The confusion matrix indicates that the model correctly classified 441 out of 457 MRI volumes, with a total of 16 misclassifications, including 6 T1-weighted images misclassified as T2-weighted and 10 T2-weighted images misclassified as T1-weighted. The classification metrics achieved on the test set resulted in an overall accuracy of 0.97, which means that the model performed well in both classes. In addition, a macro-averaged precision of 0.96, a macro-averaged recall of 0.95, and a macro average F1-score of 0.96 were obtained, confirming a balanced performance between the two classes and demonstrating that increasing the number of epochs contributed to improved accuracy. This enhancement is significant, as it indicates that the model has learned to distinguish between T1 and T2 images with greater accuracy. A weighted average precision of 0.97, weighted average recall of 0.96, and weighted average F1-score of 0.96 were obtained. This means that the model performed well in both classes because it takes into account the number of instances for each class, giving more importance to classes with more samples. This extension is justifiable in a clinical context, where maximizing the use of available information is crucial, leading to better adaptation to dataset characteristics and greater stability of the metrics. This model is therefore considered the final version for the task of automatic classification of T1- and T2-weighted MRI images on the dataset used.

Overall, these results demonstrate a significant improvement over the first trained model, achieving high levels of accuracy and stability. Both the T1 and T2 classes exhibit strong precision and recall values, confirming the model’s ability to correctly discriminate between the two MRI sequences, even when confronted with previously unseen images.

### 4.2. Segmentation of Anatomical Structures in T1 and T2 MRI

This stage employed a modular approach, training separate segmentation networks for vascular structures, abdominal organs and musculoskeletal structures, leveraging the distinct contrast characteristics of each MRI sequence (T1-weighted, T2-weighted and a combination of both sequences). Our idea is to use the sequence classification model developed in the previous stage to route each MRI volume to the most appropriate segmentation network, thereby optimizing segmentation performance based on the specific imaging characteristics. We hypothesize that this modular design will enhance segmentation accuracy by tailoring the model to the unique features of each MRI sequence. For it, three different input datasets were used for training: a combined dataset of T1- and T2-weighted images (MR), a T1-specific dataset, and a T2-specific dataset. Each segmentation network was trained independently for each anatomical block, allowing for a detailed analysis of performance across different structures and sequences. All networks were based on the 3D U-Net architecture [5] and were trained using the Dice loss function, which is particularly well-suited for medical image segmentation tasks due to its effectiveness in handling class imbalance. We compared the performance of networks trained on the combined dataset (MR) against those trained specifically on T1-weighted and T2-weighted images to assess the benefits of sequence-specific training. A disadvantage of this modular approach is the increased computational cost and complexity associated with training and maintaining multiple models. However, we believe that the potential improvements in segmentation accuracy justify this trade-off, especially in clinical applications where precision is crucial. Another disadvantage is that the specialized networks were trained on a dataset with fewer samples compared to the combined dataset, which could limit the model’s ability to generalize effectively.

**Figure 4:**
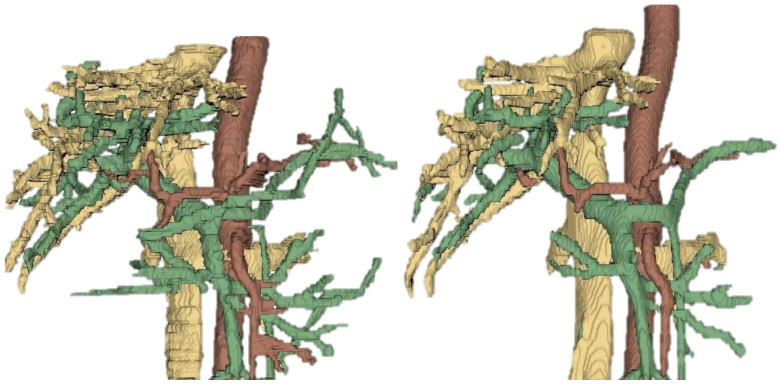
Example of vascular segmentation results on test set. (**Left**): Ground truth segmentation provided by specialists. (**Right**): Model inference output. Only very small differences can be observed between both segmentations. The segmentation model has difficulties segmenting very small vascular structures, but more large ones are very well segmented.

The first segmentation block focused on vascular structures, including the portal vein, venous system, and arterial system. This block was chosen as the initial focus due to the critical clinical importance of accurately segmenting vascular anatomy in abdominal MRI, which is essential for various diagnostic and therapeutic applications. The performance of the vascular segmentation networks was evaluated using the Dice coefficient for each structure across different input datasets. The Dice scores obtained for the vascular segmentation networks are summarized in Table 5. The T1-specific network achieved the highest overall performance, particularly for arterial and venous structures, while the T2-specific network showed strong performance for the venous system. The combined MR network provided moderate results across all structures. These findings indicate that sequence-specific networks enhance segmentation accuracy and support the design of a modular, multi-stage segmentation pipeline. The networks trained on the combined MRI dataset (MR) achieved moderate performance, with Dice scores of 0.4651, 0.7700, and 0.7023 for the portal, venous, and arterial systems, respectively. The T1-specific network showed improved segmentation for arterial structures (Dice = 0.8052) and venous structures (Dice = 0.7865), while the T2-specific network performed comparably for the venous system (Dice = 0.7904) but showed lower scores for portal and arterial vessels. Overall, the macro-average Dice (background excluded) highlights that T1-weighted images provided the most consistent performance across all vascular structures, suggesting that contrast characteristics in T1 images facilitate better discrimination of vessel boundaries in abdominal MRI. These results demonstrate the utility of specialized networks for each sequence type and inform subsequent stages of the modular segmentation pipeline. Figure 4 illustrates representative segmentation outputs and ground truth build for specialists. A simple visual inspection confirms the quantitative results, showing that the T1-specific network produces detailed segmentations of vascular structures compared to the ground truth provided by the medical specialists.

**Figure 5:**
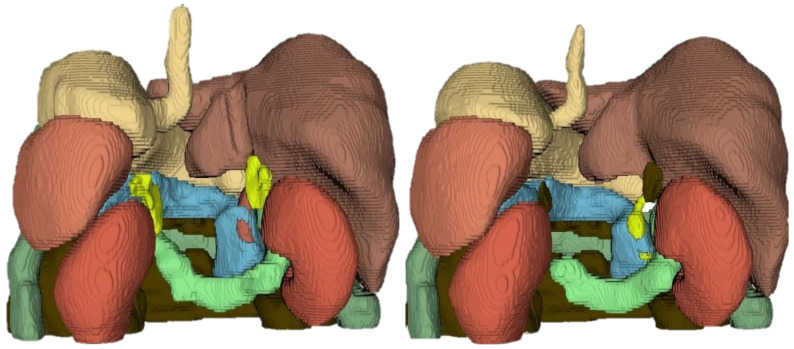
Example of abdominal organ segmentation results on test set. (**Left**): Ground truth segmentation provided by specialists. (**Right**): Model inference output. Only very small differences can be observed between both segmentations. The position of the liver, kidneys, spleen, and duodenum is well delineated with consistent accuracy between both segmentations.

The second segmentation block focused on abdominal organs, including both hollow (e.g., stomach, colon, duodenum, intestinal loops) and solid structures (e.g., liver, pancreas, kidneys, spleen). This block represents a natural extension of the vascular segmentation, since most of these organs are anatomically related to the vascular system. The performance of the abdominal organs segmentation networks was evaluated using the Dice coefficient for each structure across different input datasets. Unlike the vascular block, a label imbalance strategy was adopted, allowing training with partially annotated cases. This approach maximized dataset utilization by incorporating images with incomplete annotations, which reflects real-world clinical conditions where full segmentations are not always available. As a result, the model was exposed to a greater diversity of samples, favoring its robustness in heterogeneous scenarios. The networks were trained consistently across three different input datasets (MR, T1-weighted, T2-weighted) in a similar manner to the vascular block, ensuring methodological comparability with the vascular block. The Dice scores obtained are summarized in Table 6. Overall, the best performance was observed for large and well-defined structures such as the liver, spleen, and kidneys, which consistently achieved Dice coefficients above 0.85 in MR and T1 modalities. Medium performance was observed for organs such as the pancreas and stomach, while small or morphologically variable structures (e.g., gallbladder, bile duct, adrenal glands, intestinal loops, and tumors) showed more modest scores, reflecting the higher difficulty of their segmentation. The macro-average Dice scores across all abdominal organs (background excluded) are summarized in Table 7. The MR network achieved the highest macro Dice score of 0.678, indicating the best overall performance across the diverse set of organs. The T1-specific network followed with a macro Dice of 0.605, while the T2-specific network had the lowest score of 0.531. These results reinforce the advantage of using a combined dataset for training, as it allows the model to leverage complementary information from both T1 and T2 sequences, leading to improved segmentation accuracy across a wide range of anatomical structures. These results highlight the importance of adopting a modular segmentation approach: while robust segmentations can be obtained for major abdominal organs, specialized strategies such as data augmentation, targeted pretraining, or cascaded refinement may be required to improve performance in smaller or less well-defined anatomical structures. Figure 5 illustrates representative segmentation outputs for abdominal organs, comparing the model’s inference with the ground truth annotations. The visual results demonstrate the model’s ability to accurately delineate major organs such as the liver and kidneys, while also highlighting areas for improvement in smaller or more complex structures.

**Figure 6:**
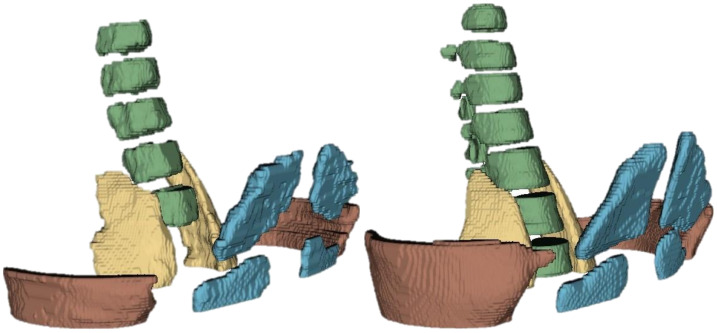
Example of musculoskeletal segmentation results on test set. (**Left**): Ground truth segmentation provided by specialists. (**Right**): Model inference output. Some big differences can be observed between both segmentations. The position of the spine, pelvis, and femurs is well delineated with consistent accuracy between both segmentations.

The third segmentation block focused on musculoskeletal structures, including both osseous (spine, pelvis, femurs) and muscular tissues (psoas, obliques, rectus abdominis, obturator internus, pyramidal muscles). This block was selected due to the clinical relevance of accurately segmenting musculoskeletal anatomy in abdominal MRI, which is essential for various diagnostic and therapeutic applications. The performance of the musculoskeletal segmentation networks was evaluated using the Dice coefficient for each structure across different input datasets. The performance of the musculoskeletal segmentation networks is summarized in Table 8. For osseous structures, the networks trained on T2-weighted images achieved the highest Dice coefficients overall, with values of near 0.77 for the pelvis, 0.80 for the spine, and 0.88 for the femurs. These results highlight the advantage of the T2 contrast in delineating cortical bone and surrounding connective tissue. By contrast, MR- and T1-based networks exhibited moderate performance, particularly in the segmentation of the spine, where Dice scores did not exceed 0.63. For muscular structures, the results were more heterogeneous. While large muscles such as the psoas achieved robust Dice scores across modalities (0.76–0.78), smaller or thinner structures, such as the rectus abdominis and the pyramidal muscles, showed more variable outcomes, with values ranging between 0.53 and 0.83. This variability reflects the intrinsic difficulty of segmenting small-volume structures, which are prone to annotation inconsistencies and partial-volume effects. Overall, these results indicate that musculoskeletal segmentation benefits substantially from T2-weighted acquisitions, particularly for osseous structures. For muscular tissues, however, performance appears to be less dependent on modality and more affected by structural variability and annotation quality. These findings reinforce the importance of multimodal integration and data augmentation strategies as potential avenues for improvement in subsequent iterations of the pipeline. The macro-average Dice coefficients (background excluded) for the musculoskeletal segmentation networks are summarized in Table 8. The T2-weighted network achieved the highest macro Dice score of 0.7544, indicating superior overall performance across both bone and muscle structures. The T1-weighted network followed with a macro Dice of 0.7027, while the MR network had the lowest score of 0.6611. These results underscore the effectiveness of T2-weighted imaging for musculoskeletal segmentation tasks, particularly in capturing the complex anatomy of both osseous and muscular tissues. Figure 6 illustrates representative segmentation outputs for musculoskeletal structures, comparing the model’s inference with the ground truth annotations. The visual results demonstrate the model’s ability to accurately delineate major structures such as the spine and femurs, while also highlighting areas for improvement in smaller or more complex muscular tissues.

### 4.3. Results of the Integrated Pipeline and Qualitative Validation

**Figure 7:**
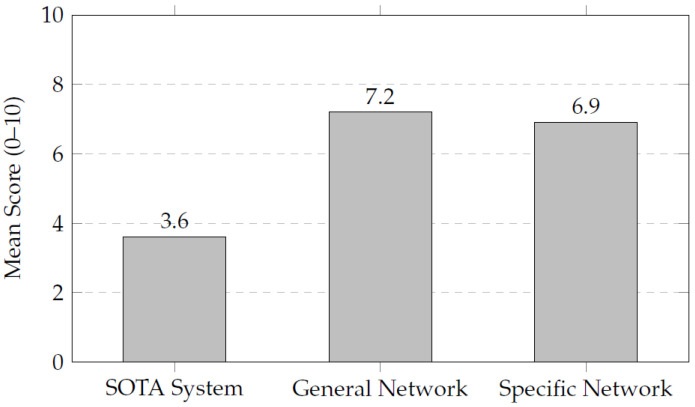
Comparison of mean qualitative scores obtained by the state-of-the-art (SOTA) system, the General Network, and the Specific Network. The proposed networks significantly outperform the commercial solution, demonstrating their superior anatomical fidelity and clinical usefulness.

**Figure 8:**
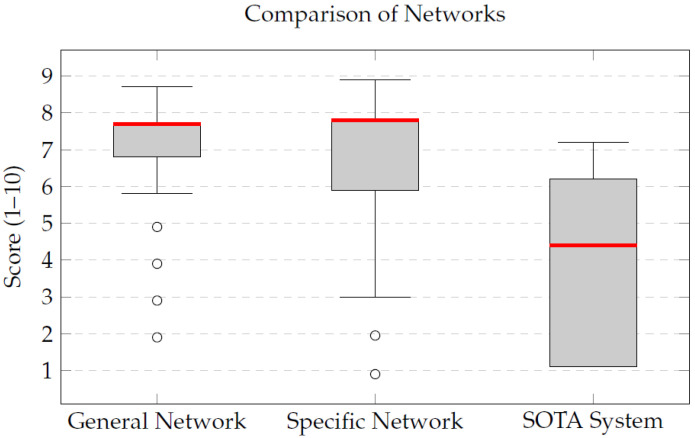
Comparison of median qualitative scores obtained by the SOTA system, the General Network, and the Specific Network using box-and-whisker plots. The red line indicates the median value for each network. The proposed Specific Network significantly outperforms the commercial and General Network solutions, demonstrating superior anatomical fidelity and clinical usefulness.

After integrating the different classification and segmentation blocks into an automated pipeline, a qualitative clinical validation was conducted in order to assess the clinical applicability of the proposed models. Eight independent studies (four T1-weighted and four T2-weighted MRIs), unseen during training and testing, were evaluated. Each case was segmented using three distinct approaches: a state-of-the-art segmentation pipeline based on TotalSegmentator, the General Network (trained jointly on T1 and T2 images), and the Specific Network (trained separately for each modality). A blinded assessment was performed by an experienced radiologist, who scored the anatomical fidelity and clinical interpretability of the segmentations on a scale from 0 to 10. The results indicate that the proposed networks provided higher qualitative scores than the previously deployed workflow for the evaluated cases. Table 9 provides an overview of the mean qualitative scores obtained for each patient. The General Network achieved an average of 7.2, closely followed by the Specific Network with 6.9, while the state-of-the-art TotalSegmentator framework obtained a mean score of 3.6. This indicates that the integrated pipeline produces anatomically consistent segmentations with strong clinical usefulness for the evaluated set of examples. Figure 7 visually summarizes these results, illustrating the difference in the qualitative assessments between the proposed networks and the earlier workflow. The analysis of median values per patient, as shown in Table 9 and Figure 8, further supports this observation, with the General Network obtaining a global median of 7.7, very close to the Specific Network (7.8), while TotalSegmentator showed a median score of 4.1. All experiments were performed using an NVIDIA RTX 3060 GPU (NVIDIA Corporation, Santa Clara, CA, USA).

These results reinforce the robustness of the proposed pipeline, particularly highlighting the ability of the General Network to achieve stable performance across both T1- and T2-weighted images without the need for modality-specific training. Overall, this qualitative validation demonstrates that the proposed system provides high-quality segmentations suitable for supporting personalized surgical planning. In summary, the quantitative analysis of the segmentation performance, as measured by Dice coefficients, further corroborates the advantages of the Specific Network, which consistently outperformed the General Network across various organs. The results reveal the possibility of using classification and segmentation models in an integrated pipeline as an ensemble to improve the accuracy and efficiency of segmentation in MRI. The qualitative validation of the integrated pipeline demonstrates its clear superiority over existing commercial solutions. The General Network, in particular, stands out for its ability to deliver high-quality segmentations across different MRI modalities, making it a versatile and effective tool for clinical applications. These promising results pave the way for further development and eventual clinical integration of the proposed system.

The qualitative evaluation was conducted by a single experienced radiologist. While this provided expert clinical insight into the anatomical fidelity and usability of the segmentations, inter-rater variability was not assessed in this study. As a result, the qualitative scores should be interpreted as an initial expert validation rather than a statistically robust inter-observer analysis.

## 5. Discussion

The qualitative evaluation of the integrated segmentation pipeline revealed notable improvements when compared to a state-of-the-art segmentation framework, TotalSegmentator. The General Network, trained on a combined dataset of T1- and T2-weighted MRIs, achieved a mean score of 7.2, demonstrating its capability to generalize effectively across different imaging modalities. This versatility is particularly advantageous in clinical settings where both types of sequences are commonly used.

Despite being trained with less than half the number of cases used for the General Network, the Specific Network achieved higher qualitative scores, with a median of 7.8. This result showed higher median scores than the General Network and also outperformed the state-of-the-art TotalSegmentator framework. This trend was observed across both T1 and T2 images, highlighting the robustness of the specialized models.

The discrepancy between the mean (6.9) and the median (7.8) for the Specific Network indicates that, although a few cases received lower evaluations, the majority of the segmentations were of high and homogeneous quality. This reinforces the added value of training specialized models tailored to each MRI sequence, which appear to capture sequence-specific morphological and contrast characteristics more effectively. Conversely, the state-of-the-art TotalSegmentator framework yielded lower mean and median scores in the evaluated examples, reflecting reduced anatomical fidelity for these specific cases.

Beyond the qualitative assessment, a complementary volumetric analysis was conducted to compare the segmented organ volumes across the different networks, as summarized in Table 10. Results revealed that the Specific Network generally produced slightly larger segmentations compared to the General Network. In practical terms, the specialized model tended to generate more complete anatomical masks, potentially reflecting an increased sensitivity for detecting and delineating structures; however, this behavior may also indicate a propensity toward over-segmentation in some cases. Consequently, volumetric metrics should be interpreted alongside qualitative clinical evaluations, which ultimately guide model usability in real-world surgical planning scenarios.

To facilitate the clinical integration of the proposed pipeline, the segmented models were incorporated into Cella’s 3D Planner, a web-based visualization platform used in clinical practice. This tool enables interactive inspection of the reconstructed anatomy directly in modern web browsers. Clinicians can rotate, zoom, and selectively display or hide structures, enhancing anatomical understanding and procedure preparation.

**Figure 9:**
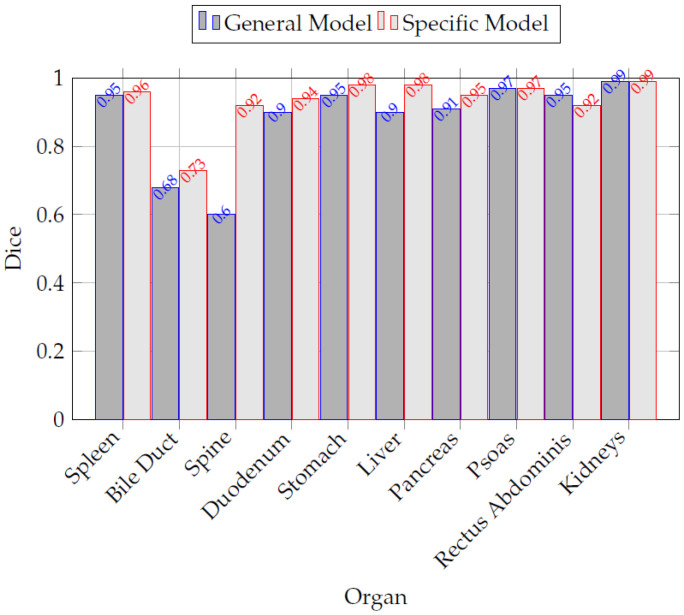
Comparison of Dice scores between the General and Specific Networks for various organs. The Specific Network consistently outperforms the General Network, particularly in challenging structures such as the bile duct and duodenum.

Table 10 presents an extract of the volumetric results, illustrating these differences across representative anatomical structures. Figure 9 compares the Dice scores obtained by the General and Specific Networks for various organs. The Specific Network showed higher Dice scores across most structures, particularly in challenging anatomies such as the bile duct and duodenum. This performance difference underscores the advantage of training specialized models for each MRI sequence, which can better capture the unique anatomical and contrast characteristics present in T1- and T2-weighted images. The enhanced segmentation accuracy achieved by the Specific Network translates into more consistent anatomical representations for the evaluated structures. Our work highlights the growing synergy between imaging technologies and artificial intelligence. We leverage deep learning architectures such as ResNet for classification and nnU-Net for segmentation to automate the analysis of sequence-related information. This AI-driven approach improves the efficiency of image interpretation and exemplifies the potential of intelligent processing systems in medical imaging, enabling more informed clinical decisions and personalized patient care for the evaluated cases.

## 6. Limitations and Future Work

Despite the promising results obtained in this study, several limitations must be acknowledged. First, the qualitative clinical evaluation was conducted on a limited subset of eight cases and assessed by a single experienced radiologist. While this provided valuable expert insight into anatomical fidelity and clinical plausibility, inter-rater reliability was not assessed. Consequently, the qualitative scores should be interpreted as an initial expert validation rather than a statistically rigorous multi-observer study. Future work will include multiple independent radiologists from different institutions to enable formal inter-rater agreement analysis using established statistical metrics.

Second, the comparison with the TotalSegmentator framework was not designed as a fully controlled benchmark. Differences in training objectives, label definitions, and optimization strategies limit the fairness of direct performance comparisons. Therefore, the reported results should be interpreted as a practical reference against an existing automated pipeline rather than as definitive evidence of methodological superiority.

Third, although specific efforts were made to curate and balance the available datasets across MRI sequences, segmentation performance may still be influenced by residual heterogeneity in label availability across anatomical structures. While data selection and preprocessing strategies were applied to maximize consistency and data usage, an exhaustive per-structure label distribution analysis was not the primary focus of this study. As a result, subtle performance differences observed between MR, T1, and T2 networks should be interpreted with caution. Future studies will incorporate more detailed reporting of label distributions and structure-specific sample sizes to further strengthen result interpretability.

In addition, the current classification pipeline lacks ablation studies and explainability analyses. Techniques such as Grad-CAM will be explored to improve transparency and to assess whether the model relies on meaningful anatomical contrast patterns or on spurious imaging artifacts.

Finally, the integration of segmented anatomical models into 3D visualization platforms is supported by existing literature as a valuable tool for surgical planning, anatomical understanding, and precision surgery. Previous studies have demonstrated the clinical relevance of patient-specific 3D reconstructions across multiple surgical domains, including pancreatic, urological, oncological, and pediatric surgery [26,27,28]. While the present work focuses on the development and validation of an automated AI-based pipeline, future studies may further build upon this established evidence by incorporating task-specific clinical metrics and prospective validation scenarios.

## 7. Conclusions

This study presents a comprehensive deep learning-based pipeline for the classification and segmentation of abdominal MRI scans, specifically targeting T1- and T2-weighted sequences. The proposed system demonstrates consistent qualitative and quantitative performance across the evaluated cases, particularly in terms of anatomical fidelity and segmentation quality.

The main objective of this work—the development of an artificial intelligence-based tool capable of automatically classifying magnetic resonance (MR) images into T1 and T2 sequences and segmenting relevant anatomical structures for oncology-oriented surgical planning research—has been successfully achieved.

The main contributions include the construction of a curated clinical MRI dataset, the integration of automatic sequence classification using a ResNet18-based model achieving over 90% accuracy, and the implementation of sequence-specific segmentation networks based on the nnU-Net v2 architecture.

The modular design of the proposed pipeline enables flexible extension, supports block-wise evaluation of anatomical structures, and facilitates scalable research-oriented MRI analysis workflows.

Overall, this work establishes a technically robust and extensible framework for sequence-aware MRI classification and segmentation, providing a solid foundation for further research in AI-assisted medical image analysis.

## Figures and Tables

**Table 1:** Distribution of the dataset used in the classification task, showing the number of T1 and T2 volumes in each training stage.

Dataset	T1 MRI	T2 MRI	Total
Proof of concept	174	189	363
Amplied filter	1588	698	2286

**Table 2:** Selected anatomical structures for modular segmentation, grouped by clinical and anatomical relevance.

Category	Anatomical Structures
Vascular	Portal vein and branches; hepatic artery and bifurcations;
	hepatic veins; inferior vena cava
Abdominal	Digestive: colon (ascending, transverse, descending, sigmoid);
	stomach; duodenum; small bowel loops
	Parenchymal: liver; pancreas (head, body, tail); kidneys (cortex,
	medulla); spleen; gallbladder; common bile duct; adrenal glands;
	tumor (pathological)
Musculoskeletal	spine; pelvis; femurs; psoas muscles; oblique muscles;
	rectus abdominis; obturator internus; pyramidalis

**Table 3:** nnU-Net-based segmentation network architecture (3D).

Component	Details	Activation/Notes
Input	3D patch from MRI volume	-
Encoder	Multiple 3D convolutions per level, downsampling at each stage	ReLU, BatchNorm
Decoder	Multiple 3D convolutions per level, upsampling at each stage	ReLU, BatchNorm
Skip Connections	Between encoder and decoder levels	Concatenation
Output Layer	Number of channels = number of structures	Softmax

**Table 4:** Comparison of classification reports across the three training stages on the test set (T1 vs. T2). Metrics show a consistent improvement through training stages.

		Precision	Recall	F1-Score	Support
First Stage	T1	0.94	0.83	0.88	41
	T2	0.87	0.96	0.91	50
	Accuracy			0.90	91
	Macro avg	0.91	0.89	0.90	91
	Weighted avg	0.91	0.90	0.90	91
Second Stage	T1	0.97	0.97	0.97	318
	T2	0.92	0.94	0.93	139
	Accuracy			0.96	457
	Macro avg	0.95	0.95	0.95	457
	Weighted avg	0.96	0.96	0.96	457
Third Stage	T1	0.97	0.98	0.98	318
	T2	0.96	0.93	0.94	139
	Accuracy			0.96	457
	Macro avg	0.96	0.95	0.96	457
	Weighted avg	0.97	0.96	0.96	457

**Table 5:** Dice scores for vascular segmentation networks across different input datasets, including Macro Dice. Macro Dice is the average of Dice scores across all abdominal organs (background excluded). MR = combined T1 and T2 datasets. T1 = T1-specific dataset. T2 = T2-specific dataset.

Network	Background	Portal	Venous	Arterial	Macro Dice
MR	0.6458	0.4651	0.7700	0.7023	0.6458
T1	0.7264	0.5877	0.7865	0.8052	0.7265
T2	0.6449	0.4908	0.7904	0.6535	0.6449

**Table 6:** Dice scores for abdominal organs across different input datasets. MR = combined T1 and T2 datasets. T1 = T1-specific dataset. T2 = T2-specific dataset.

Large abdominal organs								
Network	Backg.	Colon	Stomach	Liver	Pancreas	Kidneys	Spleen	Duodenum
MR	0.6476	0.6539	0.8424	0.9322	0.7679	0.8805	0.9491	0.6251
T1	0.5812	0.5667	0.8341	0.8796	0.7700	0.8665	0.9174	0.6160
T2	0.6449	0.4908	0.7904	0.6535	0.6449	0.4908	0.7904	0.6535
Smaller abdominal structures								
Network	Gallbladder	Bile duct	Adrenals	Intestinal loops	Tumor			
MR	0.6677	0.4039	0.4836	0.4979	0.0667			
T1	0.7831	0.2230	0.2488	0.2675	0.0843			
T2	0.6449	0.4908	0.7904	0.6535	0.0762			

**Table 7:** Macro Dice scores across different input datasets. Macro Dice represents the average Dice score across all abdominal organs, excluding background.

Network	Macro Dice
MR	0.678
T1	0.605
T2	0.531

**Table 8:** Dice coefficients for the musculoskeletal block across different input datasets. Macro Dice is the average of Dice scores across all structures (background excluded). MR = combined T1 and T2 datasets. T1 = T1-specific dataset. T2 = T2-specific dataset.

		Osseus-Related Structures			Muscle-Related Structures					
Network	Backg.	Spine	Pelvis	Femurs	Psoas	Obliques	Rectus Abdominis	Obturator Internus	Pyramidal Muscles	Macro Dice
MR	0.6610	0.6120	0.7573	0.7530	0.7783	0.4959	0.5362	0.6872	0.6686	0.6611
T1	0.5902	0.6318	0.7896	0.7917	0.7780	0.5212	0.5483	0.7899	0.7708	0.7027
T2	0.7544	0.8019	0.7685	0.8812	0.7600	0.6131	0.5535	0.8220	0.8346	0.7544

**Table 9:** The left half of the table shows the mean qualitative scores (0–10) obtained for each patient and network, while the right half displays the corresponding median values.

Patient Number	SOTA System	General Network	Specific Network		SOTA System	General Network	Specific Network
#1	2.1	8.0	6.7		4.0	8.0	7.0
#2	6.4	7.3	6.0		8.0	8.0	8.0
#3	1.3	6.2	5.4		1.0	7.0	7.0
#4	3.7	7.7	7.5		4.5	8.0	8.0
#5	4.4	8.4	8.2		5.5	8.5	8.0
#6	3.5	7.4	7.6		5.0	8.0	8.0
#7	3.2	6.1	7.1		3.5	7.0	7.5
#8	2.2	6.7	6.7		1.0	7.0	8.0
Mean	3.6	7.2	6.9	Median	4.1	7.7	7.8

**Table 10:** Extract from the complete table showing the segmented volume for each network. It can be observed that the Specific Network tends to predict larger volumes.

Patient Number	Organ	Dice	General Net. Seg. Vol. (mL)	Specific Net. Seg. Vol. (mL)	Difference (mL)	Best
#1	Spleen	0.9785	217.93	211.24	6.69	General
#2	Bile Duct	0.3636	0.10	0.36	−0.26	Specific
#3	Spine	0.5929	106.51	98.10	8.41	General
#5	Duodenum	0.8287	69.32	92.02	−22.70	Specific
#1	Stomach	0.9049	98.37	109.75	−11.38	Specific
#2	Adrenal Glands	0.1107	1.35	0.96	0.39	General
#5	Liver	0.9876	2362.44	2390.30	−27.86	Specific
#4	Obliques	0.6247	35.19	47.49	−12.30	Specific
#6	Pancreas	0.9504	89.15	84.71	4.44	General
#8	Venous Vasculature	0.9321	56.57	58.22	−1.65	Specific

## Data Availability

The data supporting the findings of this study are not publicly available due to privacy and ethical restrictions related to clinical imaging data.
